# Perioperative Management of Robotic-Assisted Gynecological Surgery in a Super Morbidly Obese Patient

**DOI:** 10.7759/cureus.29674

**Published:** 2022-09-28

**Authors:** Shunsuke Noguchi, Osamu Takahata, Akira Tsukada, Mai Shimada, Nobuaki Kikuchi

**Affiliations:** 1 Anesthesiology, Sunagawa City Medical Center, Sunagawa, JPN; 2 Anesthesiology, Asahikawa Kosei General Hospital, Asahikawa, JPN

**Keywords:** morbidity, time to surgery, anesthetic management, dietary restriction, super-morbid obesity

## Abstract

We report the perioperative management of a 32-year-old woman with super-morbid obesity (body mass index (BMI) of 60.9 kilograms per meter squared (kg/m^2^)) who underwent a robotic-assisted total laparoscopic hysterectomy in a hospital that was not specialized for obese patients. She successfully reduced her BMI by 10% using dietary restrictions in five weeks, and her surgery was performed two weeks later by consultation between gynecologists and anesthesiologists. This case demonstrates that the determination of the optimal time for surgery by consultation between physicians is crucial in the care of such a complex patient in hospitals that are not specialized for morbidly obese patients. Weight reduction in the preoperative period should be considered for super-morbid obesity patients with malignant diseases, even if the duration of preoperative optimization is shorter than four to eight weeks.

## Introduction

Perioperative management of a patient with super-morbid obesity (MO), defined as having a body mass index (BMI) of ≥50 kilograms per meter squared (kg/m^2^), is challenging due to large physiological changes, especially in the respiratory system [1-3]. Laparoscopic gynecological surgeries using robotic-assisted (RA) technology have been reported to reduce postoperative morbidity in morbidly obese patients [4-6] but suggested that the steep Trendelenburg position and pneumoperitoneum required for this surgery cause deterioration in respiratory physiology [2,4,6]. High-volume centers and university hospitals report [6,7] the necessity of multidisciplinary coordination for these complex cases [5,6]. The increase in the prevalence of obesity [8] means that there will be more opportunities for perioperative management of morbidly obese patients, even in hospitals without specific experience with obese patients.

In this case report, the perioperative management of a woman with super-MO who underwent RA total laparoscopic hysterectomy (TLH) with bilateral salpingo-oophorectomy (BSO) for treatment of endometrial cancer in a facility that is not a high-volume center for obese patients is described.

## Case presentation

The patient was a 32-year-old American Society of Anesthesiologists physical status 3 female with super-MO. Her height was 157.8 cm, body weight was 151.6 kg, and BMI was 60.9, and she was diagnosed as having endometrial cancer. She had no past medical history and was referred to a gynecologist for planning for RA-TLH with BSO. There was no high-volume center for morbidly obese patients with gynecological malignant diseases on our main island, so her gynecologist decided to perform her surgery in our hospital. Fourteen days after this, she was admitted and had a checkup by her anesthesiologist. Her neck circumference was 48 cm, she could not maintain a supine position due to dyspnea, and her oxygen saturation on pulse oximetry (SpO_2_) was over 90% on room air. A respiratory function test showed that vital capacity as a percentage of predicted (%VC) was 75.6%.

After admission, a program for preoperative weight loss by dietary restriction, exercise therapy, and respiratory rehabilitation was started. The goal for reduction in BMI was set to 10% at the time of the initial referral [9]. A total energy intake starting from 1360 kcal/day was decided.

Three days after admission, the initial simulation was performed by the patient, gynecologists, anesthesiologists, and dedicated room staff in the surgical theater. Her BMI was 59.9, and she suffered from dyspnea in the supine position. Dyspnea deteriorated, and slippage was seen in the Trendelenburg position of 15º.

The target BMI was achieved five weeks after admission and preoperative dieting was determined to continue until her gynecological procedure. The patient was scheduled for surgery two weeks later by consultation between gynecologists and anesthesiologists. The second simulation, performed 39 days after admission, verified her acceptance of the respiratory condition in Trendelenburg tilt of 15º. The patient's BMI reduced to 54 kg/m^2^, and her %VC improved to 82.2%.

Arterial blood gas analysis performed seven weeks post-admission on room air, reported arterial oxygen saturation of 93.6% with a partial pressure of carbon dioxide (PaCO_2_) of 47.8 millimeters of mercury (mmHg), and a serum bicarbonate concentration of 27.9 mmol/L. Sleep studies revealed that the patient had severe obstructive sleep apnea with multiple desaturation episodes along with obesity hypoventilation syndrome. The obesity surgery mortality risk stratification (OS-MRS) [10] and the STOP-BANG screening questionnaire for obstructive sleep apnea [11] were scored as two and five, respectively.

On the day of surgery (49 days after admission), her BMI was 53.3 kg/m^2^. SpO_2_ was 94% on air. An upper body wedge was used to posture a ramped position [3], and the reverse Trendelenburg position was applied at the induction of anesthesia. Preoxygenation with 10 L/min of 100% oxygen via a face mask was conducted for 5 min. Remifentanil was dosed on lean body weight (LBW) and infused at 0.1 μg/kg/min. After the injection of 1 mg of midazolam, 8% of lidocaine was sprayed around her pharynx, larynx, and glottis using blade #3 of McGrath Mac (Covidien Japan, Tokyo, Japan) in an awakening state. A tracheal tube (internal diameter of 7.0 mm) was successfully inserted into the trachea, and 60 mg of rocuronium (ROC) and 4 mg of midazolam were injected, and inhalation of sevoflurane was started. She was ventilated mechanically with the ventilator instrumented in a Carestation 650 Anesthesia Delivery System (GE Healthcare Japan, Tokyo, Japan) using a pressure-controlled ventilation volume-guaranteed mode. Tidal volume was set at 400 mL, positive end-expiratory pressure (PEEP) was 10 cm of water (cmH_2_O), peak inspiratory pressure (P_peak_) did not exceed 35 cmH_2_O, and respiratory rate was adjusted to maintain end-tidal partial pressure of carbon dioxide (P_ET_CO_2_) within 45-55 mmHg. The inspiratory oxygen fraction was set at 0.5, and the anesthesia was maintained using sevoflurane, remifentanil, and fentanyl. Repetitive train-of-four (TOF) stimulation with the TOF-Watch SX monitoring program (MSD, Tokyo, Japan) using the corrugator supercilii muscle was performed. Ultrasound-guided subcostal transverse abdominis plane blocks were performed bilaterally.

After insertion of intra-abdominal trocars and establishment of pneumoperitoneum with 10 mmHg of insufflation pressure, the surgical procedure with a da Vinci^TM^ Robotic System (Intuitive Surgical, Inc, Sunnyvale, USA) was commenced in the Trendelenburg position of 15º. During the surgical procedure, P_ET_CO_2_ could be maintained between 41 and 46 mmHg with a respiratory rate of 13-16 breaths/min, and P_peak_ was 21 and 32 cmH_2_O in the supine and Trendelenburg position with pneumoperitoneum, respectively. Continuous infusion of ROC at 7 µg/kg of LBW/min was started at the appearance of T1. About two hours after starting the anesthetic management, the volatile anesthetic was changed to desflurane. Hyperinflation of the lungs by holding the inspiratory airway pressure at 30 cmH_2_O for five seconds was applied several times, and SpO_2_ could be maintained between 99% and 100% throughout the surgery.

Spontaneous breathing resumed 3 min after the cessation of anesthetics, and sugammadex at 2 mg/kg of real body weight was injected. After awakening and recovering from adequate spontaneous breathing, extubation was performed in the reverse Trendelenburg position. The duration of anesthesia was 304 min. Inhalation of 3 L/min of oxygen via a nasal cannula was started, and SpO_2_ was 99%. A continuous infusion of fentanyl at a dose of 25 µg/hr was started.

She was inhaling 2 L/min of oxygen via a nasal cannula with a 45º head-up tilt, and SpO_2_ was over 90% at the time of transfer to the intensive care unit. She was transferred to the general ward the next day without any complications.

## Discussion

Perioperative management of a woman with super-MO who underwent RA-TLH was achieved in a facility that is not a high-volume center for obese patients. Preoperative optimization using dietary restriction and several simulations performed by gynecologists, anesthesiologists, and operation staff were useful for achieving complex anesthetic management safely. Consultation between gynecologists and anesthesiologists was crucial to determining the duration of preoperative optimization.

Perioperative management of a patient with super-MO is challenging due to large physiological changes, especially in the respiratory system [1-3]. Guidelines for perioperative management of obese patients have been established [3,9], and preoperative optimization, ideally for four to eight weeks, is recommended to perform surgical procedures safely [9].

It has been reported that the OR-MRS score is associated with risk factors of mortality for obese patients undergoing gastric bypass surgery [10], and this would be applicable to obese patients undergoing non-bariatric surgery [3]. The mortality odds ratio for BMI ≥50 is 3.6 (the highest among variables in the score), and BMI could be the only adjustable factor for preoperative optimization. We decided, therefore, to attempt preoperative weight reduction even though our patient had a malignant disease. It has been recommended that preoperative dieting should be performed to reduce BMI by 10% or to <55 kg/m^2^ to perform laparoscopy safely [9].

The time from diagnosis to the first and definitive surgery, defined as time to surgery (TTS), has been reported to have a negative impact on overall survival in patients with several types of cancer [12-14]. A decrease in survival rate with the prolongation of TTS has also been reported for patients with endometrial cancer. TTS of more than six to eight weeks has a negative impact on overall survival in patients with endometrial cancer [15-17]. Prolongation of TTS in morbidly obese patients with endometrial cancer would improve respiratory function but worsen overall survival. TTS in the present case was nine weeks, and the period was determined by consultation between gynecologists and anesthesiologists. Shalowitz et al. [17] suggested that adequate preoperative optimization should have priority over expedited surgery. The discussion of the risk of case delay weighed against the risk of not optimizing the patient's health status should be performed between gynecologists and anesthesiologists. It has been reported that body weight can be reduced in two weeks by using a very low-calorie diet [18-20]. Weight reduction in the preoperative period should be considered for super-MO patients, even if the duration of preoperative optimization is short compared with that in the present case.

Teamwork and high-volume experience among multidisciplinary physicians and staff are necessary for achieving RA gynecological surgery in a patient with morbid obesity [5,6], and outcomes have been improved as the surgical team gains experience [5]. An increase in the prevalence of obesity has been reported [8], and there will be more opportunities for perioperative management of morbidly obese patients, even in hospitals without specific treatment for obese patients. We could not find high-volume centers for morbidly obese patients with gynecological malignancies on our main island. We, therefore, decided to perform her surgery in our hospital. Simulations were thought to have the potential for improving outcomes and reducing complications while enhancing teamwork in the present case. Good communication among all members of the team throughout the perioperative period is necessary for completing surgery safely in challenging situations [6].

## Conclusions

Preoperative optimization using dietary restriction and several simulations performed by gynecologists, anesthesiologists, and operation staff were useful for achieving the perioperative management of a patient with super-MO safely in a hospital that is not specialized for obese patients. With multidisciplinary discussion, this specific high-risk patient had a good outcome from a high-risk anesthetic and procedure. The determination of the optimal time for surgery by consultation between gynecologists and anesthesiologists is crucial in the care of such a complex patient. Weight reduction in the preoperative period should be considered for super-MO patients with malignant diseases, even if the duration of preoperative optimization is shorter than four to eight weeks.
